# Efficacy and safety of a Venus A valve among Chinese patients undergoing transcatheter aortic valve replacement: a systematic review and single-arm meta-analysis

**DOI:** 10.3389/fcvm.2026.1725106

**Published:** 2026-02-12

**Authors:** Zeyu Sun, Dongkai Shan, Jing Wang, Bo Jiang, Tao Chen, Yingqian Zhang, Tianwen Han, Weiran Wang, Jun Guo, Changfu Liu

**Affiliations:** 1 Senior Department of Cardiology, The Sixth Medical Center of PLA General Hospital, Beijing, China; 2 Department of Cardiology, The First Medical Center of PLA General Hospital, Beijing, China

**Keywords:** complications, meta-analysis, mortality, transcatheter aortic valve replacement, Venus A valve

## Abstract

**Background:**

The Venus A valve is a first-generation self-expanding valve used in China for transcatheter aortic valve replacement (TAVR). However, data on its efficacy and safety remain limited. The present study assessed the efficacy and safety of the Venus A valve in Chinese patients undergoing TAVR.

**Methods:**

A single-arm meta-analysis was performed, and relevant studies were systematically retrieved from PubMed, Embase, Web of Science, the Cochrane Library, ClinicalTrials.gov, and Google Scholar from inception until 1 June 2022. Domestic libraries were not searched due to data overlap. All study types evaluating the Venus A valve were considered for inclusion, except case reports or reviews. Non-English language studies or those without corresponding data were excluded. The Newcastle–Ottawa scale (NOS) was used to evaluate the included retrospective studies, and the methodological index for non-randomized studies (MINORS) was used to assess the included non-randomized study (single-arm study). The random-effects model was used to calculate the combined proportion and 95% confidence interval (CI) when *I*^2^ was >50%; otherwise, a fixed-effect model was used. Publication bias was assessed using the Egger test, with *P* < 0.05 indicating potential bias.

**Results:**

This meta-analysis included 15 studies involving 1,144 Chinese patients who underwent TAVR with a Venus A valve. The device/procedure success rate was 90%. The mean transvalvular gradient decreased from 58.52 to 10.85 mmHg, and the peak jet velocity decreased from 4.86 s to 2.23 m/s. At the 30-day follow-up evaluation, the all-cause mortality rate was 3%. The requirement for a second valve accounted for 12% of cases. Major vascular complications were uncommon, as were major bleeding, stroke, acute kidney injuries, and new-onset atrial fibrillation. At the 1-year follow-up evaluation, the all-cause mortality rate was 7%. The incidence of new permanent pacemaker implantation in patients with bicuspid aortic valves was 16%, and the all-cause mortality rate was 8% at the 30-day follow-up evaluation.

**Conclusions:**

Despite its relatively high requirement for a second valve, the Venus A valve is feasible for Chinese patients undergoing TAVR. Its effectiveness and safety were demonstrated by a high device/procedure success rate, immediate hemodynamic improvement, and low incidence of complications.

## Introduction

### Background

Since the introduction of transcatheter aortic valve replacement (TAVR) into global clinical practice over 20 years ago, a vast portfolio of high-quality clinical data has confirmed the efficacy and safety of TAVR across the entire surgical risk spectrum (1–3). Although TAVR development has been relatively recent in China, a rapid and comprehensive development phase began in 2017 with the approval of two domestic valves (2). The Venus A valve (Venus Medtech, Hangzhou, China) is a first-generation domestic self-expanding valve (SEV) approved by the China Food and Drug Administration. By 2015, the TAVR clinical trial involving the Venus A valve was completed in China (ClinicalTrials.gov identifier: NCT01683474), and investigators demonstrated promising results in the treatment of patients with bicuspid or tricuspid aortic valve (TAV) stenosis who were deemed unsuitable for surgery (4). The TAVR clinical trial (4) assessed the clinical profile and outcomes of TAVR in patients with severe symptomatic aortic stenosis (AS). The preliminary results implied that treatment of AS with the Venus A valve is effective.

### Rationale and knowledge gap

**Figure 1:**
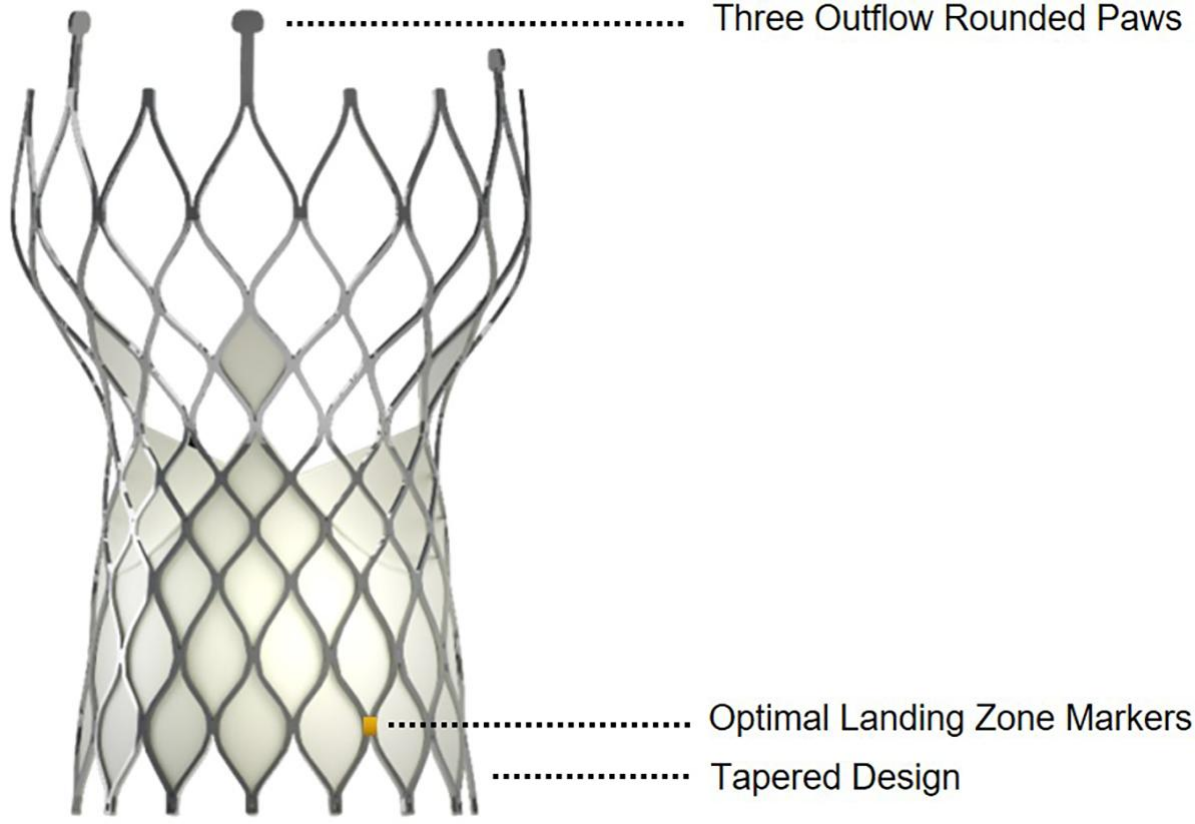
Illustration of the Venus A valve.

The Venus A valve is a self-expanding frame manufactured by suturing valve leaflets and a skirt made from a single layer of porcine pericardium into a tri-leaflet configuration (4). The first-generation SEV developed in the United States was the CoreValve transcatheter bioprosthesis (Medtronic, MN, USA) (5). The Venus A valve, like the CoreValve, has a supra-annular design with the functioning prosthetic leaflets located above the native annulus when properly deployed (4). However, the Venus A valve device differs from other valves in several respects. Specifically, there are three rounded paws rather than two at the outflow end for coaxial loading, radiopaque markers of 0.5 cells above the inflow end indicate the optimal landing zone, and the inflow end is tapered to protect the conduction tissue (Figure 1). To date, there has been limited data describing the procedural, clinical, and valve performance outcomes of the Venus A valve, the first-generation domestic SEV for TAVR in China.

### Objective

The present meta-analysis analyzed and evaluated the efficacy and safety of the Venus A valve in Chinese patients. We present this article in accordance with the PRISMA reporting checklist.

## Methods

### Search strategy, study selection, and data extraction

A systematic search was performed to retrieve relevant literature from PubMed, Embase, Web of Science, the Cochrane Library, ClinicalTrials.gov, and Google Scholar from inception until 1 June 2022. Domestic libraries were not searched due to data overlap. The search code was as follows: (“Transcatheter Aortic Valve Implantation [TAVI]” OR “Transcatheter Aortic Valve Replacement [TAVR]” OR “Percutaneous Aortic Valve” OR “Transcatheter Aortic Valve”) AND “Venus A.”

In the present study, the following inclusion criteria were used: (1) All study types except case reports or reviews were considered for inclusion (e.g., cross-sectional studies and cohort studies); (2) patients undergoing TAVR with the Venus A valve were included in the study; and (3) the study reported the procedural, clinical, and valve performance results of the Venus A valve. The following studies were excluded: (1) duplicate studies; (2) studies containing other valves, the data of which could not be extracted separately; and (3) non-English language studies or no corresponding data. Two investigators (CL and DS) independently assessed the quality of articles and extracted relevant data. Any disagreements in quality assessment were resolved through consultation with a third investigator (JG).

### Outcomes of interest

Efficacy outcome measures include the device/procedure success rate, the mean transvalvular gradient (PGmean), peak jet velocity (Vmax), and left ventricle ejection fraction (LVEF) at baseline, during hospitalization, and at the follow-up evaluation.

Safety outcomes of the Venus A valve included pre-dilation, post-dilation, conversion to surgical aortic valve replacement (SAVR), the need for a second valve, moderate or severe paravalvular leakage (PVL), and adverse events. Adverse events at the 30-day follow-up evaluation included life-threatening or disabling bleeding, major vascular complications, new permanent pacemaker implantation (PPI), acute kidney injury (AKI), new-onset atrial fibrillation (AF), stroke, and all-cause mortality. In addition, adverse events at the 1-year follow-up evaluation included all-cause mortality. Furthermore, the safety of the Venus A valve for a bicuspid aortic valve (BAV) was preliminarily examined.

The Valve Academic Research Consortium 3 (VARC-3) was used to define TAVR-specific outcomes (6), while study-specific definitions remained in the corresponding articles. The success of the device/procedure was defined as the absence of surgical mortality, accurate placement of a singular artificial valve in the appropriate anatomic position, and achieving the anticipated effect. Type 3 (life-threatening) bleeding was defined as overt bleeding occurring in a critical organ, causing hypovolemic shock or severe hypotension, or necessitating reoperation, surgical exploration, or re-intervention to achieve hemostasis. Type 4 (leading to death) bleeding was defined as any overt bleeding event that directly resulted in mortality. Major vascular complications are defined as the occurrence of aortic dissection/rupture, vascular injury, distal embolization, unplanned endovascular/surgical intervention, or closure device failure, provided that any of these events result in death, VARC type ≥2 bleeding, limb/visceral ischemia, irreversible neurologic impairment, amputation, or irreversible end-organ damage.

### Quality of assessment

The Newcastle–Ottawa scale (NOS) was used to evaluate the included retrospective studies (7). The methodological index for non-randomized studies (MINORS) was used to assess the included non-randomized study (single-arm study) (8).

### Statistical analysis

The single-arm meta-analysis was conducted using Stata statistical software (version 17.0). The original data from the literature were transformed using the double arcsine method to conform to the normal distribution before analysis using Stata. The initial meta-analysis conclusion was then restored using the following formula to make a final conclusion: *P* = [sin (tp/2)]^2^. The *I*^2^ value was used to assess heterogeneity. The random-effects model was used to calculate the combined proportion and 95% confidence interval (CI) when *I*^2^ was >50%; otherwise, a fixed-effect model was used. Publication bias was assessed using the Egger test, with *P* < 0.05 indicating potential bias.

## Results

### Search results

**Figure 2:**
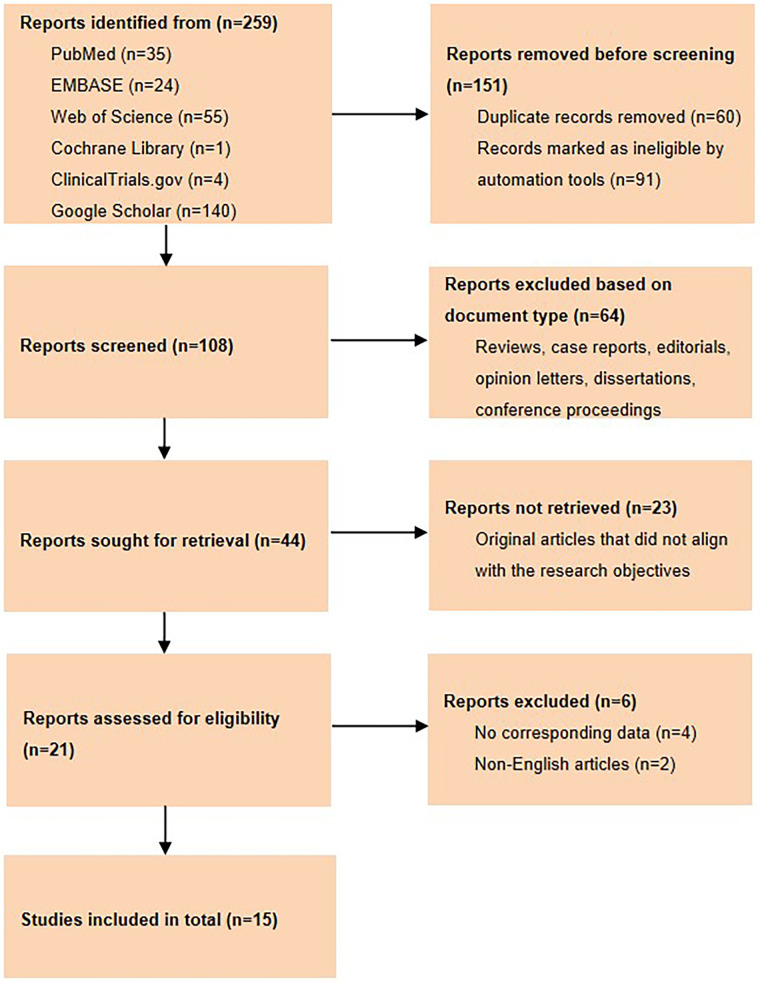
Flow chart of the study selection process.

A comprehensive search yielded a total of 259 articles, of which 151 were excluded due to duplication or automation exclusion. Additionally, 64 articles did not meet the specified inclusion criteria, 23 articles had inconsistent objectives, and 6 articles had no corresponding data or were non-English language articles and were excluded. Therefore, a total of 15 articles were deemed suitable for inclusion in the present meta-analysis, encompassing data from 1,144 Chinese patients. The Preferred Reporting Items for Systematic Reviews and Meta-Analyses (PRISMA) 2020 flow diagram depicts the study flow (Figure 2).

**Figure 3:**
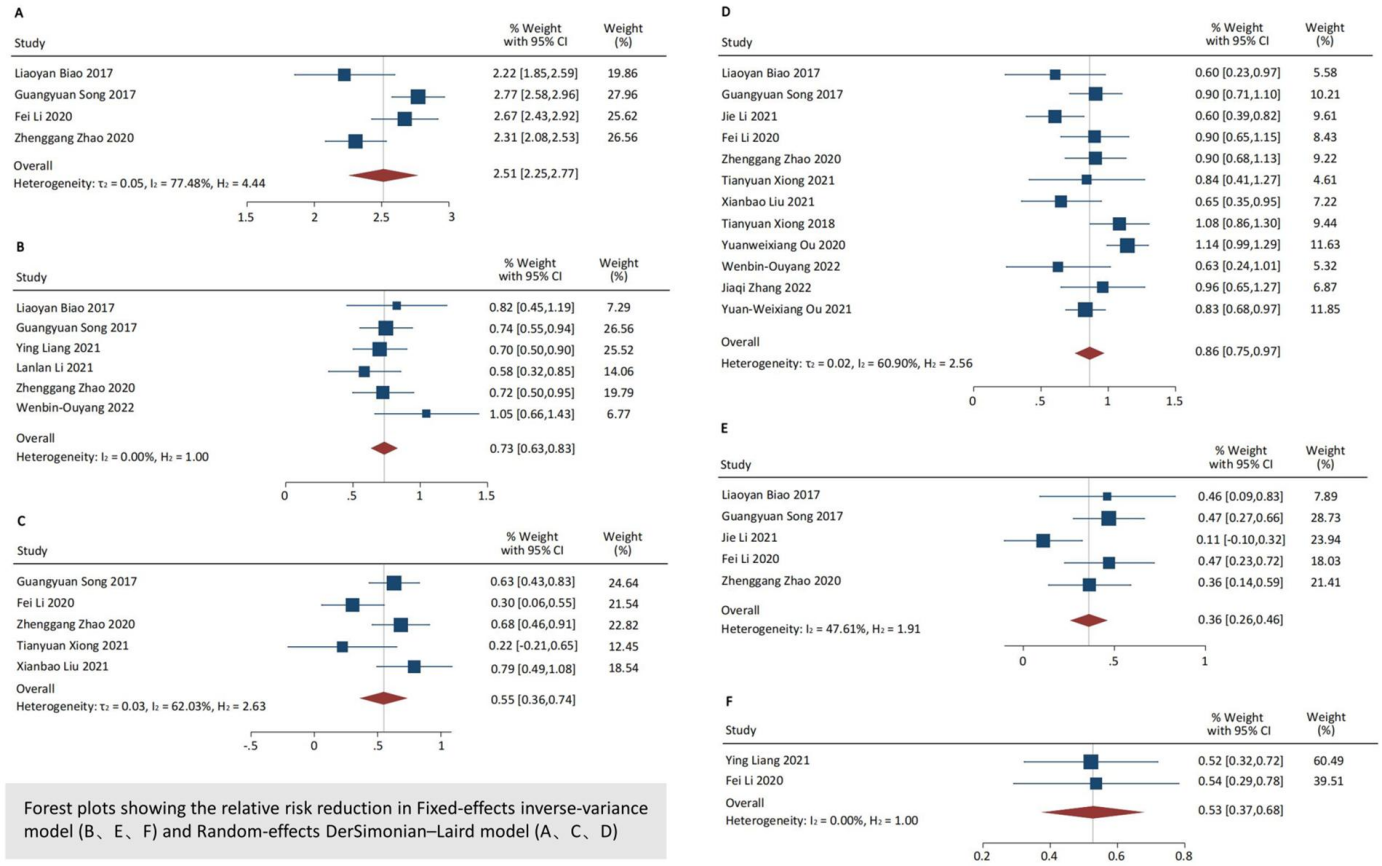
Pooled results of procedure details and adverse events within hospitalization and at follow up. Forest plots showing the relative risk reduction in Fixed-effects inverse-variance model **(B, E, F)** and Random-effects DerSimonian-Laird model **(A, C, D)**; **(A)** device/procedure success during hospitalization, **(B)** need a second valve during hospitalization, **(C)** PVL ≥ moderate at 30-day follow-up evaluation, **(D)** new PPI at 30-day follow-up evaluation, **(E)** all-cause mortality at 30-day follow-up evaluation, **(F)** All-cause mortality at 1-year follow-up evaluation.

### Details of all studies and quality assessment

All 15 eligible studies were conducted in China, including 14 retrospective studies and 1 single-arm study. Supplementary Table S1 presents the quality assessment results of the included studies. Supplementary Tables S2 and S3 present the echocardiographic characteristics and outcome indicators of the included studies, respectively. All retrospective studies had quality assessment scores ≥7. The MINORS index of the single-arm study was 10. Basic information from the studies is detailed in Table 1.

**Table 1:** Basic information from the included studies.

Study author	Year	Recruitment period	Follow-up time, months	Research patients	Patients who used Venus A	Average age, years	Gender, male	STS score, %	Study type
Liaoyan Biao	2017	2012–2014	24	54	27	74.1	15	6.2	Retrospective
Guangyuan Song	2017	2012–2015	6	101	101	75.4	58	5.5	Retrospective
Ying Liang	2021	2012–2018	12	284	97	TAVI: 76.6 SAVR: 72.7	175	–	Retrospective
Jie Li	2021	2016–2020	1	84	84	Optimal position: 74.5 Malposition: 71.3	43	–	Retrospective
Lanlan Li1	2021	2018–2019	–	53	53	68.1	42	–	Retrospective
Fei Li	2020	2014–2017	1	163	63	76.1	35	8.3	Retrospective
Zhengang Zhao	2020	2017–2018	1	75	75	73.8	44	7.4	Retrospective
Tianyuan Xiong	2021	–	–	20	20	72.5	11	–	Retrospective
Xianbao Liu	2021	–	–	43	43	BAV: 76.4 TAV: 79.4	26	–	Retrospective
Tianyuan Xiong	2018	2012–2017	–	80	56	75.0	47	–	Retrospective
Abdullah Hagar	2020	2012–2017	–	256	145	74	145	–	Retrospective
Yuanweixiang OU	2020	2015–2017	–	165	165	74	–	–	Single-arm
Wenbin Ouyang	2022	2020–2021	12	178	97	BEV: 72.4 SEV: 72.0	99	–	Retrospective
Zhangjia Qi	2022	2019–2021	1	39	39	75	21	–	Retrospective
Yuanweixiang OU	2021	2015–2019	1	181	181	Without HAVB: 72.8 HAVB: 74.1	103	–	Retrospective

STS, Society of Thoracic Surgeons; TAV, tricuspid aortic valve; SAVR, surgical aortic valve replacement; BAV, bicuspid aortic valve; SEV, self-expanding valve; HAVB, high-degree atrioventricular block.

### Baseline characteristics of patients

The baseline characteristics of patients are presented in part in Tables 1 and 2. The patients in the extractable data were nearly 70 years of age, and 61.2% were males. A 5%–10% score on the Society of Thoracic Surgeons (STS) risk calculator indicated intermediate risk. Overall, 75% of patients were in the New York Heart Association (NYHA) functional class ≥III. The overall patient disease spectrum is listed in Table 2.

**Table 2:** Baseline characteristics of the study populations.

Study author	Year	Patients	PPI	BBB	PVD	CABG	MI	PCI	Stroke	AF	CKD	CLD	Hypertension	Diabetes	CAD	NYHA Class III/IV	Postprocedural AVmax, m/s
Liaoyan Biao	2017	54	–	–	–	–	2	–	7	12	6	–	–	10	18	50	CoreValve: 2.5 ± 0.5 Venus A: 2.3 ± 0.36
Guangyuan Song	2017	101	5	13	32	2	5	9	15	15	–	40	50	17	–	80	2.2 (2.0–2.6)
Ying Liang	2021	284	–	20	52	–	22	28	100	39	–	37	109	52	–	23	–
Jie Li	2021	84	1	–	14	–	–	14	7	18	–	5	41	18	35	50	–
Lanlan Li	2021	53	–	–	–	–	–	–	–	–	–	–	–	–	–	–	–
Fei Li	2020	163	–	–	–	5	–	–	26	29	–	–	94	44	61	141	SAPIEN:2.25 ± 0.37 Venus A 2.33 ± 0.56 J-Valve 2.56 ± 0.61
Zhengang Zhao	2020	75	–	–	35	–	–	–	–	14	–	41	32	7	33	70	–
Tianyuan Xiong	2021	20	–	–	–	–	–	–	–	–	–	–	–	–	–	–	–
Xianbao Liu	2021	43	–	–	–	–	–	–	–	–	–	–	–	–	–	–	–
Tianyuan Xiong	2018	80	21	–	36	–	3	–	–	28	9	43	42	13	29	77	PPI:2.5 (2.2–2.8) No PPI:2.3 (2.0–2.6)
Abdullah Hagar	2020	256	–	–	143	–	5	–	34	37	36	162	114	46	110	234	2.44 ± 1.1
Yuanweixiang OU	2020	165	48	–	–	–	–	–	–	–	–	–	–	–	–	–	–
Wenbin Ouyang	2022	178	5	–	36	8	–	38	29	19	–	15	98	40	56	–	–
Zhangjia Qi	2022	39	–	–	12	–	–	–	13	9	–	3	–	13	23	14	–
Yuanweixiang OU	2021	181	–	–	72	–	–	–	–	–	–	84	70	35	67	158	–

PPI, permanent pacemaker implantation; BBB, bundle branch block; PVD, peripheral vascular disease; CABG, coronary artery bypass graft surgery; MI, myocardial infarction; PCI, percutaneous coronary intervention; AF, atrial fibrillation; CKD, chronic kidney disease; CLD, chronic lung disease; CAD, coronary artery disease; NYHA, New York Heart Association; AVmax, maximum velocity of aortic valve.

### Efficacy outcomes

The device/procedure success rate was 90% (95% CI: 0.81–0.96, *I*^2^ = 77.48%; Table 3). Three studies reported PGmean and Vmax improvement (4, 9, 10). The PGmean was 58.52 mmHg (95% CI: 47.24–69.79, *I*^2^ = 90.42%) and the Vmax was 4.86 m/s (95% CI: 4.72–5.00, *I*^2^ = 0.00%) prior to TAVR. The PGmean decreased to 10.85 mmHg (95% CI: 9.54–12.17, *I*^2^ = 0.00%) and the Vmax decreased to 2.23 m/s (95% CI: 2.04–2.44, *I*^2^ = 69.69%) during hospitalization (Table 4).

**Table 3:** Procedure details and adverse events during hospitalization and at the follow-up evaluation.

Variable	Results of meta-analysis	*I* ^2^	Adjusted results	Results of the Egger test, *P*
Procedure details and adverse events during hospitalization				
Device/procedure success	2.51 (95% CI: 2.25–2.77)	77.48%	0.90 (95% CI: 0.81–0.96)	0.207
Pre-dilation	2.52 (95% CI: 2.06–2.9)	85.76%	0.90 (95% CI: 0.73–0.99)	0.117
Post-dilation	1.22 (95% CI: 0.79–1.66)	83.81%	0.28 (95% CI: 0.15–0.54)	0.095
Conversion to SAVR	0.30 (95% CI: 0.11–0.50)	44.77%	0.02 (95% CI: 0.00–0.06)	0.802
Need a second valve	0.73 (95% CI: 0.63–0.83)	0.00%	0.12 (95% CI: 0.06–0.19)	0.221
PVL ≥ moderate	0.55 (95% CI: 0.36–0.74)	62.03%	0.07 (95% CI: 0.03–0.13)	0.297
Adverse events at the 30-day follow-up evaluation				
Major vascular complication	0.40 (95% CI: 0.25–0.54)	0.00%	0.03 (95% CI: 0.01–0.07)	0.740
Major bleeding	0.64 (95% CI: 0.49–0.80)	0.00%	0.09 (95% CI: 0.05–0.15)	0.977
Stroke	0.16 (95% CI: 0.04–0.28)	0.00%	0.00 (95% CI: 0.00–0.02)	0.784
AKI	0.36 (95% CI: 0.21–0.50)	0.00%	0.03 (95% CI: 0.01–0.06)	0.658
New-onset AF	0.74 (95% CI: 0.50–0.98)	42.11%	0.13 (95% CI: 0.06–0.22)	0.188
New PPI	0.86 (95% CI: 0.75–0.97)	60.90%	0.17 (95% CI: 0.13–0.21)	0.182
All-cause mortality	0.36 (95% CI: 0.26–0.46)	47.61%	0.03 (95% CI: 0.01–0.05)	0.631
Adverse events at the 1-year follow-up evaluation				
All-cause mortality	0.53 (95% CI: 0.37–0.68)	0.00%	0.07 (95% CI: 0.03–0.11)	0.923
Adverse events of BAV at the 30-day follow-up evaluation				
New PPI	0.83 (95% CI: 0.62–1.04)	0.00%	0.16 (95% CI: 0.09–0.25)	0.678
All-cause mortality	0.58 (95% CI: 0.33–0.83)	0.00%	0.08(95% CI: 0.03–0.16)	0.811

BAV, bicuspid aortic valve; SAVR, surgical aortic valve replacement; PVL, paravalvular leakage; PPI, permanent pacemaker implantation; AKI, acute kidney injury; AF, atrial fibrillation.

**Table 4:** Hemodynamic changes.

Variable	Results of meta-analysis	*I* ^2^	Results of the Egger test, *P*
Baseline			
PGmean, mmHg	58.52 (95% CI: 47.24–69.79)	90.42%	0.005
Vmax, m/s	4.86 (95% CI: 4.72–5.00)	0.00%	0.645
LVEF, %	56.88 (95% CI: 54.33–59.44)	11.79%	0.183
During hospitalization			
PGmean, mmHg	10.85 (95% CI: 9.54–12.17)	0.00%	0.960
Vmax, m/s	2.23 (95% CI: 2.04–2.44)	69.69%	0.827
LVEF, %	58.76 (95% CI: 56.34–61.17)	0.00%	0.776
At the 30-day follow-up evaluation			
PGmean, mmHg			
Vmax, m/s	2.25 (95% CI: 2.15–2.35)	0.00%	0.365
LVEF, %	59.93 (95% CI: 57.72–62.13)	15.60%	0.276

PGmean, mean transvalvular gradient; Vmax, peak jet velocity; LVEF, left ventricular ejection fraction.

### Safety outcomes

Eight studies have investigated the TAVR procedure details with the Venus A valve (4, 9–15). The incidence of pre- and post-dilation was 90% (95% CI: 0.73–0.99, *I*^2^ = 85.76%) and 28% (95% CI: 0.15–0.54, *I*^2^ = 83.81%), respectively. The conversion to SAVR rate was 2% (95% CI: 0.00–0.06, *I*^2^ = 44.77%), and the requirement for a second valve accounted for 12% of cases (95% CI: 0.06–0.19, *I*^2^ = 0.00%). During hospitalization, 7% of patients (95% CI: 0.03–0.13, *I*^2^ = 62.03%) had moderate or severe PVL (Table 3).

Fourteen studies reported adverse outcomes (Figure 3; Table 3) (4, 9, 10, 15–23). At the 30-day follow-up evaluation, 3% of the patients (95% CI: 0.01–0.07, *I*^2^ = 0.00%) had a major vascular complication, and 9% (95% CI: 0.05–0.15, *I*^2^ = 0.00%) had major bleeding. No strokes were reported (95% CI: 0.00–0.02, *I*^2^ = 0.00%). The incidence of AKI and new-onset AF was 3% (95% CI: 0.01–0.06, *I*^2^ = 0.00%) and 13% (95% CI: 0.06–0.22, *I*^2^ = 42.11%), respectively. The incidence of new PPI was 17% (95% CI: 0.13–0.21, *I*^2^ = 60.90%). The 30-day all-cause mortality rate was 3% (95% CI: 0.01–0.05, *I*^2^ = 47.61%). At the 1-year follow-up evaluation, the all-cause mortality rate increased to 7% (95% CI: 0.03–0.11, *I*^2^ = 0.00%).

Three studies reported adverse outcomes of the Venus A valve for BAV (4, 16, 18). The incidence of new PPI was 16% (95% CI: 0.09–0.25, *I*^2^ = 0.00%), and the all-cause mortality rate was 8% (95% CI: 0.03–0.16, *I*^2^ = 0.00%) at the 30-day follow-up evaluation (Table 3).

### Publication bias

The present study used Egger's test to detect publication bias. There was no significant publication bias in the assessment indicators (*P* > 0.05; Tables 3, 4) except for the PGmean at baseline (*P* = 0.005).

## Discussion

### Key findings

The present study evaluated the efficacy and safety of the first-generation domestic SEV (the Venus A valve) that is used for TAVR in China. The device/procedure success rate of TAVR using the Venus A valve was relatively high. Patients experience significant PGmean and Vmax hemodynamic improvements. Importantly, using the Venus A valve for TAVR did not significantly increase the complication and adverse event rates.

### Strengths and comparison with similar research

Previous studies reported that the 30-day follow-up evaluation major vascular complication, life-threatening or disabling bleeding, stroke, AKI, and new-onset AF rates with the CoreValve were 8.2%, 12.7%, 2.3%, 1.7%, and 12.9%, respectively (23–25). In the present study, the incidence of major vascular complications (3%), major bleeding (9%), stroke (0%), AKI (3%), and new-onset AF (13%) was basically consistent with previous studies at the 30-day follow-up evaluation (24, 25). These data revealed that the Venus A valve is a suitable choice for Chinese patients undergoing TAVR.

At the 30-day and 1-year follow-up evaluations, the all-cause mortality rates were 3% and 7%, respectively. Popma et al. (24) reported that the all-cause mortality rates at the 30-day and 1-year follow-up evaluations in patients with severe AS at extreme risk for surgery who received the CoreValve were 8.4% and 24.3%, respectively. In addition, Adams et al. (26) reported that the all-cause mortality rate at the 1-year follow-up evaluation in patients with severe AS who received the CoreValve at increased risk for surgery was 14.2%. The SURTAVI study (25), which used the CoreValve or Evolut R (Medtronic), demonstrated that the all-cause mortality rates at the 30-day and 1-year follow-up evaluations in patients with severe AS at intermediate risk for surgery were 2.8% and 8.1%, respectively. Therefore, the all-cause mortality rate at 30-day and 1-year follow-up in the present study was comparable to the SURTAVI study (25) but lower than the rates reported by Popma et al. (24) and Adams et al. (26). These promising results may be attributed to the near 50% prevalence of BAV in the Chinese population presenting for TAVR, which exceeds the 2%–10% overall prevalence reported in Western TAVR series (27). Patients with BAVs are younger on average than patients with TAVs (28). The potential impact of this finding is that patients referred for TAVR in China are much younger, with a mean age of 74 years. Nevertheless, the mean age of patients in European and US TAVR registries is >80 years (29). Therefore, from a safety perspective, TAVR using the Venus A valve may demonstrate equal safety to the CoreValve.

In the present study, the incidence of moderate or severe PVL during hospitalization was 7%, which was less than reported by Popma et al. (24) (10.7%). Balloon pre-dilation is a routine procedure; whether or not post-dilation is performed depends on valve performance and paravalvular regurgitation. Pre- and post-dilation facilitates delivery of the transcatheter valve and expansion of the nitinol prosthesis frame to achieve the desired hemodynamics and minimize PVL (30). The incidence of pre- and post-dilation in the current study was 90% and 28%, respectively, which are comparable to the Popma et al. (24) results. The post-dilation rate was nearly the same as the CoreValve but a lower PVL rate than the CoreValve occurred, which demonstrates that the Venus A valve exhibits comparable efficacy to the CoreValve. However, a head-to-head comparison is warranted to confirm this speculation.

The need for a second valve accounted for 12% of all cases, which was higher than reported by Popma et al. (24) (3.5%) and the Italian CoreValve Registry (3.6%) (31). The increased requirement for a second valve could be due to the following reasons: (1) TAVR was introduced relatively late in China, and the TAVR procedure requires extensive learning that was obtained from the experiences reported by other countries (32), and most cases occurred between 2012 and 2018, indicating that early TAVR procedures in China were performed with limited experience. Nevertheless, there has been a downward trend in the incidence of requiring a second valve (10, 33). (2) The relatively higher incidence of BAV was an influencing factor because prosthetic valve function could theoretically be jeopardized by pre-existing abnormal cusp fusion, heavily calcified leaflets, and an asymmetric BAV raphe (34). A higher valve-in-valve (VIV) bailout procedure was reported in the BAV cohort compared with the TAV group by De Biase et al. (35) (11% vs. 3%). 3) There were no retrievable or repositionable functions on the first-generation valves. Therefore, the valve expansion accuracy in the early phase was lower than that of the current valve prosthesis implantations. A meta-analysis revealed that the Medtronic second-generation Evolut R valve had a lower incidence of incorrect prosthetic positioning or unintended prosthetic performances than the CoreValve (3.5% vs. 5.2%) (36). It should be noted that despite the increased frequency of the VIV procedure, the clinical and echocardiographic endpoints compared favorably to those of patients undergoing the standard procedure (31).

Compared with surgery, TAVR with a self-expanding supra-annular bioprosthesis is associated with a lower incidence of disabling stroke, AKI, bleeding events, and new-onset AF, but a higher incidence of aortic regurgitation and permanent pacemaker use (37). The incidence of new PPI was 17%, which was lower than reported by Popma et al. (24) (21.6%) and the SURTAVI study (25.9%) (25), but comparable to Adams et al. (26) (19.8%) and Popma et al. (37) (17.4%). Conduction abnormalities are common after TAVR and may result in PPI (38), which is a main complication of TAVR that includes adverse effects involving left ventricular function, heart failure, and late mortality among low-risk patients with a potential for increased longevity (39). The anatomic proximity of the atrioventricular conduction system to the aortic valvular complex contributes to cardiac conduction disturbances (38). Conduction disturbances can develop due to direct physical damage or compression, such as injuries from wire manipulation or balloon aortic valvuloplasty, or compression by the valve frame, which causes ischemic damage to the conduction system cells or interstitial edema (40, 41). The shape and height of the frame may be key factors underlying this abnormality (42). We attribute this finding to the tapered design (Figure 1) at the inflow end to protect conduction tissue. Therefore, the incidence of new PPI was lower for the Venus A valve than in previous studies.

Previous guidelines and an expert consensus have identified BAV as a relative contraindication to TAVR (32). The present study generated a few preliminary results because the number of studies that have focused on BAV is limited, and few data could be extracted. Himbert et al. (43) showed that the 30-day combined safety endpoint rate in high-risk patients with stenotic BAV using the CoreValve was 93% with a PPI required in 40% of the patients. Because of the small sample size, the previous research only indicated that the Venus A valve is feasible for BAV, and the new PPI and all-cause mortality rates were not discussed in detail. Therefore, the safety and effectiveness of BAV should be investigated with a large sample size in a randomized controlled trial.

## Study limitations

The present study had several limitations. First, high heterogeneity existed in more than one-half of the outcomes. However, the complete data were rarely accessed for subgroup analysis to reduce heterogeneity. Second, there may have been differences in postoperative efficacy between the Venus A valve and the CoreValve due to the different application times and the main adaptation population. Moreover, because single-arm trials lack control groups, comparisons between the Venus A valve and the CoreValve were based on data from the population with a discrepant baseline. Therefore, it is essential to design a prospective study to explore the clinical effects of the two types of valves. Third, BAV vs. TAV could not be analyzed due to the lack of original data. Therefore, outcomes for BAV intervention were preliminary findings awaiting further verification. Finally, due to the short-term follow-up of the included studies, the long-term prognosis of the Venus A valve has not been established. Future evaluation of the long-term prognosis of this valve will be beneficial for further improving valve equipment and enhancing implantation techniques.

## Conclusions

In conclusion, this single-arm meta-analysis demonstrated that the Venus A valve is feasible for Chinese patients undergoing TAVR despite its relatively high requirement for a second valve. Moreover, preliminary findings indicated that TAVR with the Venus A valve may be suitable for Chinese patients with BAV.

### Implications and actions needed

The Venus A valve effectiveness was demonstrated by the high device/procedure success rate and immediate hemodynamic improvement. The low incidence of complications confirmed the safety of the Venus A valve. Venus A valve may be worth promoting in the application of Chinese patients. However, it is essential to design a prospective study to explore the clinical effects of two or more types of valves with subgroup analysis. Future evaluation of the long-term prognosis of this valve will be beneficial for further improving valve equipment and enhancing implantation techniques.

## Data Availability

The original contributions presented in the study are included in the article/supplementary material, further inquiries can be directed to the corresponding author/s.
