# Supplementary material for: Efficacy and safety of a Venus A valve among Chinese patients undergoing transcatheter aortic valve replacement: a systematic review and single-arm meta-analysis
Source: Front Cardiovasc Med. 2026 Feb 12;13:1725106. doi: 10.3389/fcvm.2026.1725106 (PMC12935943; doi:10.3389/fcvm.2026.1725106)
Supplement: Supplementary file 1 [file Table1.docx]

**Table S2 Characteristics of echocardiography**

| Study Author | Year | BAV | Baseline | | | | | Within Hospitalization | | | | 30-Day Follow-up | | |
| --- | --- | --- | --- | --- | --- | --- | --- | --- | --- | --- | --- | --- | --- | --- |
|  |  |  | Mixed Valve Diseases | P_mean_ | V_max_ | LVEF |  | P_mean_ | V_max_ | LVEF |  | P_mean_ | V_max_ | LVEF |
| Liaoyan Biao | 2017 | 32/54 | 7 | 59.1± 17.5 | 4.80± 0.8 | 58.1±15.0 | | 10.9±4.7 | 2.12±0.46 | 59.3±11.6 | | 12.1±4.1 | 2.3±0.36 | 61.6±9.3 |
| Guangyuan Song | 2017 | 44/97 | 26 | 59.0 (45.0–71.0) | 5.0 (4.4–5.4) | 58 (46–65) | | - | - | - | | 10.0 (7.0–13.0) | 2.3 (2.0–2.5) | 61 (56–65) |
| Ying Liang | 2021 | - | - | - | - | - | | - | - | - | | - | - | - |
| Jie Li | 2021 | - | Optimal Position:  25/61  Malposition:  11/23 | Optimal Position: 58.09 ± 19.58  Malposition:  53.03 ± 23.45 | - | - | | - | - | - | | - | - | - |
| Lanlan Li | 2021 | - | - | 70.1 ± 35.9 | - | - | | 6.6 ± 7.2 | - | - | | - | - | - |
| Fei Li | 2020 | 33/163 | - | SAPIEN:  49.57 ± 7.20  Venus-A:  52.11 ± 7.05  J-Valve:  51.37 ± 7.46 | SAPIEN:  4.56 ± 0.53  Venus-A:  4.88 ± 0.64  J-Valve:  4.75 ± 0.62 | SAPIEN: 58.81 ± 10.81  Venus-A: 55.31 ± 12.26  J-Valve: 57.94 ± 11.76 | | SAPIEN: 46.89 ± 7.08  Venus-A: 49.90 ± 7.87  J-Valve: 48.82 ± 7.26 | SAPIEN: 2.25 ± 0.37  Venus-A: 2.33 ± 0.56  J-Valve: 2.56 ± 0.61 | SAPIEN: 60.57 ± 9.89  Venus-A: 58.53 ± 11.37  J-Valve: 60.38 ± 9.43 | | SAPIEN: 44.78 ± 6.39  Venus-A: 48.70 ± 7.35  J-Valve: 48.46 ± 6.00 | SAPIEN: 2.18 ± 0.35  Venus-A: 2.21 ± 0.52  J-Valve:  2.61 ± 0.59 | SAPIEN:  61.75 ± 8.91  Venus-A:  59.17 ± 9.10  J-Valve:  62.51 ± 7.23 |
| Zhengang Zhao | 2020 | 8/75 | - | 67.6 ± 19.7 | - | 52.0 ± 16.1 | | - | - | - | | - | - | - |
| Tianyuan Xiong | 2021 | 10/20 | - | - | - | - | | - | - | - | | - | - | - |
| Xianbao Liu | 2021 | 26/43 | - | BAV: 57.7 ± 16.8  TAV: 52.9 ± 11.5 | BAV: 4.80 ± 0.92  TAV: 4.70 ± 0.42 | BAV: 55.0 ± 13.3  TAV: 54.8 ± 17.3 | | - | - | - | | - | - | - |
| Tianyuan Xiong | 2018 | 80/80 | - | New PPI:  61.2 ± 15.4  No PPI:  67.0 ± 21.3 | New PPI:  4.9 ± 0.7  No PPI:  5.1 ± 0.8 | BAV: 55.0 ± 13.3  TAV: 54.8 ± 17.3 | | - | - | - | | - | - | - |
| Abdullah Hagar | 2020 | 142/256 | - | 64.32 ± 19.3 | 5.15 ± 0.75 | 54.7 ± 15 | | - | - | - | | - | - | - |
| Yuanweixiang OU | 2020 | - | - | - | - | - | | - | - | - | | - | - | - |
| Wenbin Ouyang | 2022 | 87/178 | - | BEV：56.7±16.7  SEV：48.0±18.6 | - | BEV：62.2±10.6  SEV：56.6±13.1 | | BEV：14.3±6.1  SEV：10.8±4.9 | - | - | | - | - | - |
| Zhangjia Qi | 2022 | - | - | 49.85 ± 21.54 | - | 51.92 ± 13.05 | | - | - | - | | - | - | - |
| Yuanweixiang OU | 2021 | - | - | Without HAVB：  65.3 ± 21.5  HAVB：  62.3 ± 19.4 | Without HAVB：  5.1 ± 0.8  HAVB：  5.0 ± 0.8 | Without HAVB：  54.8 ± 15.8  HAVB：  55.2 ± 14.6 | | - | - | - | | - | - | - |

Mixed valve disease, concomitant with other valve diseases of moderate or higher severity; BEV=balloon-expanding valve; HAVB= high-degree atrioventricular block; BAV=bicuspid aortic valve; SAVR=surgical aortic valve replacement; PVL=paravalvular leakage; PPI=permanent pacemaker implantation; AKI=acute kidney injury; AF=atrial fibrillation; PGmean=mean transvalvular gradient; Vmax=peak jet velocity; LVEF= left ventricle ejection fraction

Data are presented as mean ± standard deviation.
