# Supplementary material for: Efficacy and safety of a Venus A valve among Chinese patients undergoing transcatheter aortic valve replacement: a systematic review and single-arm meta-analysis
Source: Front Cardiovasc Med. 2026 Feb 12;13:1725106. doi: 10.3389/fcvm.2026.1725106 (PMC12935943; doi:10.3389/fcvm.2026.1725106)
Supplement: Supplementary file 2 [file Table2.docx]

**TABLE S3 Outcome indicators reported in each study**

| Study Author | Year | Within Hospitalization | | | | | | 30-Day Follow-up | | | | | | | 1-Year Follow-up | |
| --- | --- | --- | --- | --- | --- | --- | --- | --- | --- | --- | --- | --- | --- | --- | --- | --- |
|  |  | Pre-dilation | Post-dilation | SAVR | VIV | PVL | Device/procedural Success | PPI | Vascular Complication | Major Bleeding | Stroke | AKI | AF | Death | Death |  |
| Liaoyan Biao | 2017 | √ | √ | √ | √ | - | √ | √ | √ | √ | √ | - |  | √ | - |  |
| Guangyuan Song | 2017 | √ | √ | - | √ | √ | √ | √ | - | - | √ | √ | - | √ | - |  |
| Ying Liang | 2021 | - | - | - | √ | √ | - | - | - | - | - | - | - | - | √ |  |
| Jie Li | 2021 | - | - | - | - | - | - | √ | √ | √ | √ | √ | √ | √ | - |  |
| Lanlan Li | 2021 | - | - | - | √ | - | - | - | - | - | - | - | - | - | - |  |
| Fei Li | 2020 | √ | √ | √ |  | √ | √ | √ | - | - | - | - | - | √ | √ |  |
| Zhengang Zhao | 2020 | - | - |  | √ | √ | √ | √ | √ | √ | √ | - | - | √ | - |  |
| Tianyuan Xiong | 2021 | √ | √ | - | - | √ | - | √ | - | - | - | - | - | - | - |  |
| Xianbao Liu | 2021 | - | - | - | - | √ | - | √ | - | - | - | - | - | - | - |  |
| Tianyuan Xiong | 2018 | - | - | - | - | - | - | √ | - | - | - | - | - | - | - |  |
| Abdullah Hagar | 2020 | √ | √ | - | - | - | - | - | - | - | - | - | - | - | - |  |
| YuanWeixiang OU | 2020 | - | - | - | - | - | - | √ | - | - | - | - | - | - | - |  |
| Wen-Bin Ou-Yang | 2022 | √ | √ | √ | √ | - | - | √ | - | - | - | - | - | - | - |  |
| Zhangjia Qi | 2022 | - | - | - | - | - | - | √ | - | - | - | - | - | - | - |  |
| Yuan-Weixiang OU | 2021 | - | - | - | - | - | - | √ | - | - | - | - | - | - | - |  |

VIV=valve in valve; PPI= permanent pacemaker implantation; BBB=bundle branch block; PVD=peripheral vascular disease; CABG=coronary artery bypass graft surgery; MI=myocardial infarction; PCI=percutaneous coronary intervention; AF= atrial fibrillation ; CKD=chronic kidney disease; CLD=chronic lung disease; CAD=coronary artery disease; NYHA=New York Heart Association
