# Supplementary material for: Efficacy and safety of a Venus A valve among Chinese patients undergoing transcatheter aortic valve replacement: a systematic review and single-arm meta-analysis
Source: Front Cardiovasc Med. 2026 Feb 12;13:1725106. doi: 10.3389/fcvm.2026.1725106 (PMC12935943; doi:10.3389/fcvm.2026.1725106)
Supplement: Supplementary file 3 [file Table3.docx]

**Table S1 Quality assessment results of including studies**

| A. Newcastle-Ottawa Scale for Included Retrospective Studies | | | | | | | | | | |
| --- | --- | --- | --- | --- | --- | --- | --- | --- | --- | --- |
| Study Author | Year | Selection | | | | Comparability | Outcome | | | Quality Score |
|  |  | Adequate Definition of Cases | Representativeness of Cases | Selection of Controls | Definition of Controls | Comparability of Cases and Controls | Assessment of outcome | Was follow-up long enough for outcomes to occur | Non-response proportion |  |
| Liaoyan Biao | 2017 | ★ | ★ | ★ | ★ | ★★ | ★ | ★ | - | 8 |
| Guangyuan Song | 2017 | ★ | ★ | ★ | ★ | ★ | ★ | ★ | - | 7 |
| Ying Liang | 2021 | ★ | ★ | ★ | ★ | ★★ | ★ | ★ | - | 8 |
| Jie Li | 2021 | ★ | ★ | ★ | ★ | ★ | ★ | ★ | - | 7 |
| Lanlan Li | 2021 | ★ | ★ | ★ | ★ | ★ | ★ | ★ | - | 7 |
| Fei Li | 2020 | ★ | ★ | ★ | ★ | ★ | ★ | ★ | - | 7 |
| Zhengang Zhao | 2020 | ★ | ★ | ★ | ★ | ★★ | ★ | ★ | - | 8 |
| Tianyuan Xiong | 2021 | ★ | ★ | ★ | ★ | ★ | ★ | ★ | - | 8 |
| Xianbao Liu | 2021 | ★ | ★ | ★ | ★ | ★★ | ★ | ★ | - | 8 |
| Tianyuan Xiong | 2018 | ★ | ★ | ★ | ★ | ★ | ★ | ★ | - | 7 |
| Abdullah Hagar | 2020 | ★ | ★ | ★ | ★ | ★★ | ★ | ★ | - | 8 |
| Wenbin Ouyang | 2022 | ★ | ★ | ★ | ★ | ★★ | ★ | ★ | - | 8 |
| Zhangjia Qi | 2022 | ★ | ★ | ★ | ★ | ★ | ★ | ★ | - | 7 |
| Yuanweixiang OU | 2021 | ★ | ★ | ★ | ★ | ★ | ★ | ★ | - | 7 |
| B. MINORS Index for Included Non-Randomized Study | | | | | | | | | | |
| Study Author | Year | I | II | III | IV | V | VI | VII | VIII | Total |
| Yuanweixiang Ou | 2020 | 2 | 2 | 2 | 2 | 2 | 0 | 0 | 0 | 10 |

Numbers I-VIII in heading signified: I, a clearly stated aim; II, inclusion of consecutive patients; III, prospective collection of data; IV, endpoints appropriate to the aim of the study; V, unbiased assessment of the study endpoint; VI, follow-up period appropriate to the aim of the study; VII, loss of follow-up less than 5%; VIII, prospective calculation of the study size. MINORS, methodologic index for non-randomized studies.
